# Inflammatory memory and comorbidities

**DOI:** 10.1002/ctm2.984

**Published:** 2022-07-15

**Authors:** George Hajishengallis, Triantafyllos Chavakis

**Affiliations:** ^1^ Department of Basic and Translational Sciences, School of Dental Medicine University of Pennsylvania Philadelphia Pennsylvania USA; ^2^ Institute for Clinical Chemistry and Laboratory Medicine, University Hospital and Faculty of Medicine Technische Universität Dresden Dresden Germany

1

Recent advances have established that the inflammatory adaptation of haematopoietic stem and progenitor cells (HSPCs) towards myeloid‐biased haematopoiesis constitutes a main driver of long‐lived trained innate immunity. The latter represents an epigenetically based memory state that leads to elevated inflammatory preparedness to future infectious or inflammatory challenges.^1^ This inflammatory memory is passed on epigenetically from HSPCs to progeny myeloid cells (‘trained’ or hyper‐responsive neutrophils and monocytes). Although trained immunity can mediate enhanced protection against subsequent infections and tumours,^1^ in a different context, such as chronic inflammatory disorders, trained innate immune cells might contribute to exacerbation of the pathology rather than provide protection (termed ‘maladaptive’ trained immunity). A few years ago, we reasoned that systemic inflammation and inflammation‐adapted HSPCs and their trained progeny may engage in a self‐sustained feed‐forward loop that may underlie the chronicity of major inflammatory disorders.^2^ Moreover, implicit in that hypothesis was the notion that maladaptively trained HSPCs may constitute a common mechanistic basis for the association of different comorbidities. In other words, inflammation‐adapted HSPCs could initiate a cascade of events that causally and reciprocally link comorbidities via a ‘trained’ inflammatory process occurring in the bone marrow (Figure 1). We have recently tested this hypothesis in the context of the periodontitis–arthritis comorbidity axis,^3^ two distinct but epidemiologically strongly associated inflammatory bone loss disorders, which lead to progressive erosion of alveolar and joint bone, respectively.^4^

**FIGURE 1:**
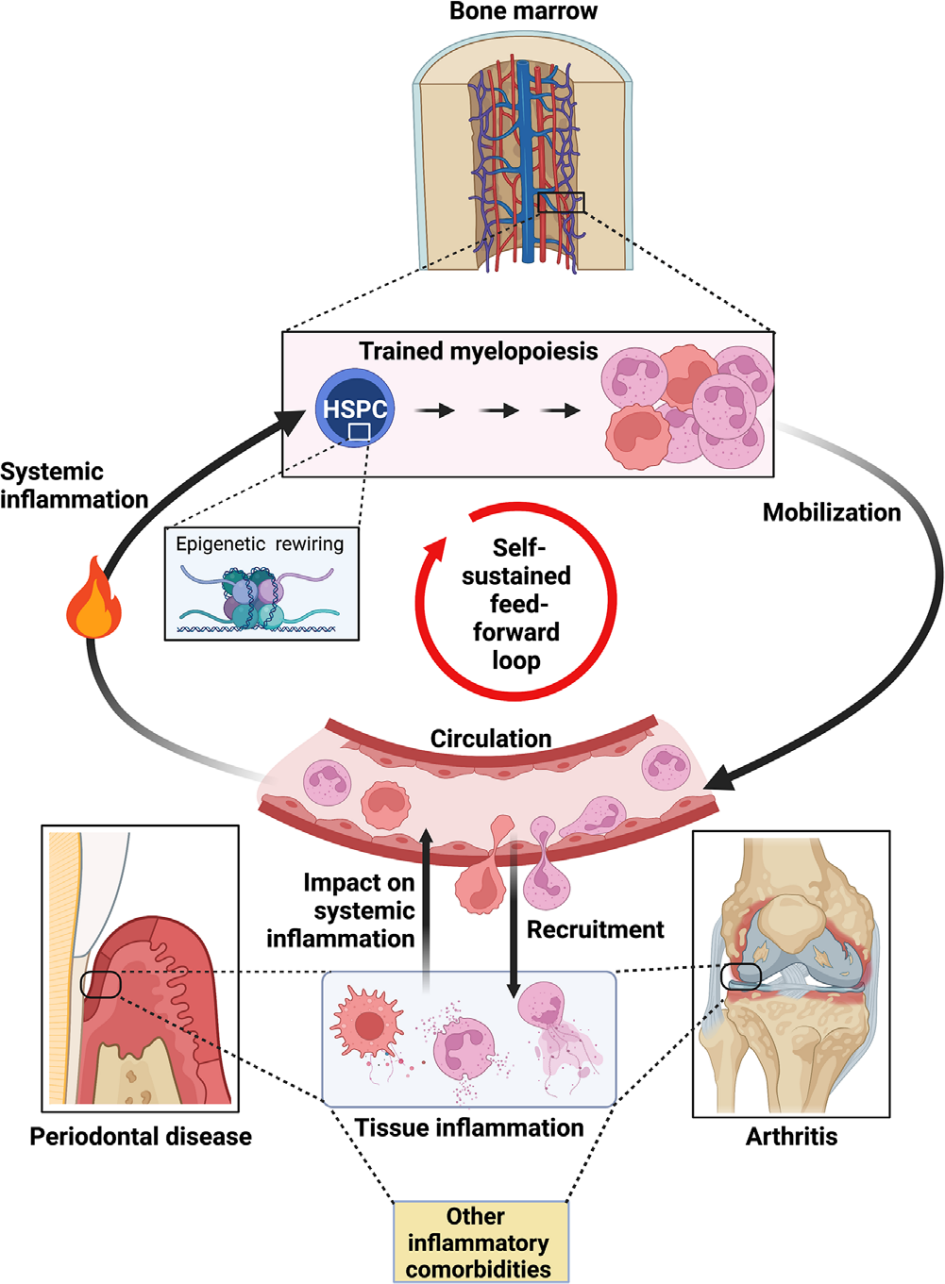
Reciprocal regulation loop between trained myelopoiesis in the bone marrow and chronic inflammatory comorbidities. Haematopoietic stem and progenitor cells (HSPCs) in the bone marrow epigenetically memorize systemic inflammation and engage in increased production of myeloid cells with enhanced inflammatory preparedness (trained myelopoiesis). As a consequence of the enhanced output of trained inflammatory myeloid cells, an existing inflammatory disease may be exacerbated or become chronic whereas the risk for another (comorbidity) may be increased. The resulting enhancement of tissue inflammation provides further stimulus for trained myelopoiesis while perpetuating the generated vicious cycle. This concept was experimentally demonstrated for periodontitis and arthritis^3^ but likely applies to additional chronic inflammatory disorders.

Using clinically relevant mouse models of periodontitis and arthritis, we showed that systemic inflammation associated with either condition could modulate HSPCs towards trained myelopoiesis. Consistently, bone marrow transplantation from periodontitis‐ or arthritis‐trained mice to naïve recipients increased the susceptibility of the latter to either disease when recipient mice were appropriately challenged 12 weeks post‐transplantation, hence, suggesting transmission of sustained inflammatory memory of haematopoietic stem cells.^3^ Indeed, systemic inflammation associated with periodontitis caused innate immune training of HSPCs (myeloid‐differentiation bias and generation of hyperresponsive myeloid cells) that was predominantly evident at the epigenetic level. This periodontitis‐induced maladaptive training of myelopoiesis failed in mice with HSPC‐specific deletion of the IL‐1 receptor and, consequently, transplantation of bone marrow from these donors to recipient mice could not transmit trained myelopoiesis and enhanced disease susceptibility in the latter.^3^

Consistent with the connection of chronic inflammation and trained myelopoiesis, numerous studies indicate that patients with chronic inflammatory conditions (e.g. periodontitis or cardiovascular disease) have elevated counts of peripheral blood myeloid cells that may exhibit hyperresponsive phenotypes, for instance, as assessed upon ex vivo stimulation, hence suggestive of inflammatory memory (reviewed in Ref. [^5^]). This enhanced hyperresponsiveness is also seen in volunteers treated with agonists of trained immunity, such as the bacillus Calmette–Guérin (BCG) vaccine, and was attributed to trained myelopoiesis associated with BCG‐induced epigenetic changes imprinted in HPSCs.^6^ Moreover, clinical imaging based on positron emission tomography/computed tomography with 2‐deoxy‐2‐[fluorine‐18] fluoro‐d‐glucose (^18^F‐FDG‐PET/CT) has revealed that inflammation in the periodontal tissue correlates positively with haematopoietic tissue activity in the bone marrow, as well as with arterial wall inflammation and risk of future cardiovascular events.^7^, ^8^ Similar ^18^F‐FDG‐PET/CT‐based correlations of elevated bone marrow haematopoietic metabolic activity and heightened arterial wall inflammation were observed in rheumatoid arthritis patients in clinical remission.^9^ Together, these findings portray the operation of a bidirectional inflammatory axis, between the bone marrow and inflamed tissues, which links distinct comorbidities. In other words, they are consistent with the concept that maladaptive trained immunity could be a common and unifying mechanism that drives or exacerbates inflammatory comorbidities (Figure 1).

Our finding that HSPCs with an epigenetically imprinted myeloid bias constitute the central hub of maladaptive trained immunity underlying distinct comorbidities has several clinical implications, assuming that a similar phenomenon operates in humans. First, it suggests that clinicians should consider assessing, in prospective clinical studies, the potential role of inflammatory memory in the donor's haematopoietic stem cells and whether it may influence outcomes in recipients of therapeutic bone marrow transplantation. For instance, to address whether this is indeed a significant issue, the transmissibility of inflammatory memory to recipients of haematopoietic stem cell transplants from donors, with or without a recent history of inflammatory disorders, could be interrogated. The observation that maladaptive trained myelopoiesis was crucially dependent on IL‐1 signalling in HSPCs suggests that systemic neutralization of IL‐1 might offer a therapeutic option for holistic treatment of inflammatory comorbidities. As atherosclerosis is also likely exacerbated by maladaptive trained immunity, one could speculate that the protective effects of antibody‐mediated neutralization of IL‐1β in the CANTOS trial for the treatment of atherosclerosis^10^ might also include the inhibition of IL‐1‐dependent trained myelopoiesis in the bone marrow.

The preclinical and clinical evidence discussed before suggests that systemic inflammation leads to epigenetic reprogramming of HSPCs towards trained myelopoiesis with the potential to perpetuate and exacerbate inflammation and generate a feed‐forward loop linking bone marrow and inflammatory comorbidities (Figure 1). A critical question that has not been adequately addressed yet involves the duration of the bone marrow–based inflammatory memory. This is important to know because, as long as maladaptive inflammatory memory exists, patients in remission or after successful treatment could conceivably still maintain an increased risk for a comorbid condition.

## CONFLICT OF INTEREST

The authors declare no potential conflict of interest.
